# Young pediatric surgeons without endoscopic surgical skill qualification safely perform advanced endoscopic surgery under the supervision of expert qualified surgeons

**DOI:** 10.1007/s00464-025-11657-w

**Published:** 2025-03-20

**Authors:** Kazuki Ota, Takahisa Tainaka, Akinari Hinoki, Chiyoe Shirota, Satoshi Makita, Akihiro Yasui, Yoichi Nakagawa, Daiki Kato, Takuya Maeda, Hiroki Ishii, Hiroo Uchida

**Affiliations:** Department of Pediatric Surgery, Nagoya University Graduate School of Medicine, 65 Tsurumai-Cho, Showa-Ku, Nagoya, 466-8550 Japan

**Keywords:** Young surgeons, Endoscopic surgical skill qualification, Minimally invasive surgery, Choledochal cyst, Biliary atresia, Lobectomy

## Abstract

**Background:**

Young pediatric surgeons should receive adequate training in various minimally invasive surgeries (MIS). However, it is essential to maintain patient safety and outcomes during the learning process. In Japan, the endoscopic surgical skill qualification (ESSQ) system in pediatric surgery was initiated by the Japan Society for Endoscopic Surgery (JSES) in 2009 to objectively evaluate the skill of endoscopic surgeons. This study compared perioperative outcomes between pediatric surgeons with ESSQ qualifications and those without (non-ESSQ).

**Methods:**

We retrospectively reviewed the medical records of children with choledochal cyst (CC), lobectomy, and biliary atresia (BA) who underwent MIS at our hospital from 2013 to 2023. We assessed the performance of nonqualified surgeons assisted by supervisors with ESSQ qualifications.

**Results:**

This study included the records of 101 surgical cases with CC (operated by ESSQ: 36, non-ESSQ: 65), 78 patients with BA (operated by ESSQ: 35, non-ESSQ: 43), and 67 patients with lobectomy (operated by ESSQ: 31, non-ESSQ: 36). For the CC and lobectomy groups, there were no significant differences in operating time, blood loss, and postoperative complications (PO) between the ESSQ and non-ESSQ groups. In BA, the operative times for the ESSQ and non-ESSQ groups were 310 and 377 min, respectively (*p* = 0.001). Furthermore, no significant differences were observed in blood loss, PO, jaundice-clearance ratio, and jaundice-free survival rate with the native liver between the two groups.

**Conclusion:**

The results indicate that young pediatric surgeons can safely perform MIS while practicing under the supervision of experienced ESSQ-qualified surgeons.

Although the birth rate is rapidly declining in Japan, the number of pediatric surgical cases has decreased by approximately 10% over the past five years [1]. Skilled pediatric surgeons with experience in many cases are critical for the future of pediatric surgical patients. Minimally invasive surgery (MIS), which preserves function with smaller incisions, is becoming increasingly used in pediatric surgery. MIS is especially significant for children who will live with the outcomes for many years. However, because endoscopic surgery is basically a solo endeavor, it demands more advanced skills and techniques from pediatric surgeons [2]. Therefore, the training of young pediatric surgeons is an extremely important issue.

In Japan, to ensure the safety and appropriateness of MIS, the endoscopic surgical skill qualification (ESSQ) system in pediatric surgery was established in 2009 by the Japan Society for Endoscopic Surgery [3]. This certification requires pediatric surgical specialty certification and emphasizes the acquisition of both general and specialized skills for comprehensive patient care. The ESSQ certifies that the surgeon meets established criteria to perform safe, advanced procedures using specialized endoscopic instruments and to mentor junior surgeons. This promotes the healthy dissemination and advancement of endoscopic surgery, ultimately contributing to public welfare. Therefore, all pediatric surgeons involved in endoscopic surgery are encouraged to obtain this certification. As of January 2025, only 72 surgeons have obtained this certification, with a pass rate of approximately 44%. The examination, which costs 30,000 Japanese yen (approximately 200 U.S. dollars), is administered annually and may be retaken multiple times without any penalties for failure [4].

The number of pediatric surgical patients in Japan is few and continues to decline each year. For example, 129 cases of esophageal atresia, 114 cases of biliary atresia (BA), and 178 cases of choledochal cyst (CC) are reported in 2021 [1]. Despite the decreasing number of patients, experienced surgeons in many centers operate on these patients, making it challenging for young pediatric surgeons to develop their surgical skills [5]. Therefore, providing adequate training to young surgeons without compromising patient outcomes is essential. Unlike laparotomy, MIS has a relatively short history and lacks extensive examination to determine whether young pediatric surgeons can safely perform MIS while learning.

This study investigated whether young pediatric surgeons without ESSQ, under the supervision of ESSQ-qualified surgeons, can perform advanced pediatric endoscopic surgery for BA, CC, and lobectomy with the same perioperative results as pediatric surgeons with ESSQ in Japan.

## Materials and methods

This single-center retrospective cohort study used data from the Department of Pediatric Surgery at Nagoya University Hospital, one of the largest pediatric surgical facilities in Japan, from 2013 to 2023. The study protocol was approved by the Nagoya University Ethics Committee (2023–0476). We conducted the study in accordance with the ethical standards established by the 1964 Declaration of Helsinki and its subsequent amendments or comparable ethical standards. We identified cases of BA, CC, and lobectomy performed via MIS at our department and retrospectively reviewed demographic data, surgeon data, and intraoperative and postoperative outcomes. We assessed the operative time, intraoperative blood loss, postoperative complications, and postoperative hospital stay. Cases of conversion to open surgery were excluded from statistical analysis. Postoperative complications were defined as Clavien–Dindo classification III or higher. For BA, jaundice-clearance was defined as a decrease in total bilirubin levels below 1.5 mg/dL.

To be eligible for ESSQ certification in Japan, applicants must be board certified in pediatric surgery and have experience with at least 20 relatively complex Group A endoscopic procedures (such as fundoplication, splenectomy, anorectal malformation repair, mediastinal tumor resection, and lung resection), with a total of over 50 endoscopic surgeries. Additionally, applicants are required to attend designated seminars and have at least two publications related to endoscopic surgery. Applicants must submit an unedited video of either a laparoscopic fundoplication or a laparoscopic splenectomy. Two experts on the JSES Committee independently assess the surgeon’s endoscopic technique based on strict criteria, which include port insertion, instrument handling, exposure of the surgical field, scope manipulation, collaboration with assistants, use of energy devices, dissection and identification of the diaphragmatic crura, securing the abdominal esophagus, preservation of the vagus nerve, the length and tightness of the wrap, and suturing techniques. Certification is awarded to those deemed to have ample skill, with each component evaluated according to a detailed scoring system, for a total of 100 points and a passing score of 75 points or higher. Therefore, ESSQ-certified surgeons are considered highly proficient. Additionally, the surgeon must have performed a minimum of 50 advanced endoscopic procedures that meet the criteria of the JSES Committee. In this study, we compared the surgical outcomes of ESSQ-qualified surgeons with those of non-ESSQ-qualified surgeons to evaluate differences in skill. The date of certification for each surgeon was identified, and the surgeon was considered qualified after the date of certification and unqualified before that date. Notably, all surgeries performed by non-ESSQ-qualified surgeons were conducted under the supervision of ESSQ-qualified surgeons, who directly participated as the primary assistant and provided real-time guidance throughout the procedure.

Statistical analyses were performed using SPSS (version 29.0). Continuous data were presented as median and range and categorical data were presented as frequency and percentage. The Mann–Whitney *U* test was used to evaluate group differences, and Fisher’s exact test was used to compare categorical variables between groups. Statistical significance was determined by a *p* value of less than 0.05.

## Results

This study included a cohort of pediatric patients who underwent MIS for BA, CC, and lobectomy at our institution, totaling 78 BA cases (ESSQ: 35, non-ESSQ: 43), 101 CC cases (ESSQ: 36, non-ESSQ: 65), and 67 lobectomy cases (ESSQ: 31, non-ESSQ: 36). During this period, these procedures were performed by 22 surgeons, 6 of whom had ESSQ certification; two of the six had ESSQ before the study period and four became ESSQ-certified during the study period.

Table 1 summarizes the comparison between ESSQ and non-ESSQ groups for BA. There were no cases of conversion to open surgery. ESSQ-qualified surgeons demonstrated significantly shorter operative times compared to non-ESSQ-qualified surgeons (310 min vs. 377 min, *p* = *0.001*). However, no statistically significant differences were observed in associated anomalies (2.8% vs. 7.0%, *p* = *0.623*), intraoperative blood loss (25.0 ml vs. 19.0 ml, *p* = *0.180*), postoperative complication rate (11.4% vs. 9.3%, *p* = *1.000*), jaundice-clearance ratio (77.1% vs. 67.4%, *p* = *0.450*), or 1-year native liver survival (77.1% vs. 72.0%, *p* = *0.795*) between the two groups. ESSQ surgeons had significantly more years of experience than non-ESSQ surgeons (25 years vs. 10 years, *p* < *0.001*). The mean number of surgeries performed by ESSQ surgeons during the study period was 8.0, while that of surgeries performed by non-ESSQ surgeons was 2.0.

**Table 1:** Demographics and operative outcomes of biliary atresia patients undergoing surgery performed by qualified and nonqualified surgeons based on the JSES Endoscopic Surgical Skill Qualification System

Factors	ESSQ	Non-ESSQ	*p value*
Patients (n)	35	43	
Conversion to open surgery (n)	0	0	
Male/Female (n/ n)	9/26	11/32	*0.989*
Body weight at operation (kg)*	4.24 [3.90–4.91]	4.30 [3.70–4.70]	*0.345*
Associated anomalies (n)	1 (2.8%) Malrotation	3 (7.0%) Malrotation, Cardiac anomaly with malrotation, portosystemic shunt and intestinal duplication with malrotation	*0.623*
Blood loss (ml)*	25.0 [10.0–43.0]	19.0 [7.0–35.0]	*0.180*
Operative time (min)*	310 [285–366]	377 [320–420]	***0.001***
Postoperative complication (n)	4 (11.4%)	4 (9.3%)	*1.000*
Jaundice-clearance ratio (n)	27 (77.1%)	29 (67.4%)	*0.344*
1-year native liver survival (n)	27 (77.1%)	31 (72.0%)	*0.611*
Years of experience (y)	25 [20–28]	10 [6–19]	***<0.001***

^*^Median (range)

Bold font indicates statistically significant difference

Table 2 summarizes the comparison between ESSQ and non-ESSQ groups for CC. There were no cases of conversion to open surgery. Patients in the ESSQ group were significantly heavier and older than those in the non-ESSQ group (17.6 kg vs. 12.2 kg, *p* = *0.046*; 4 years vs. 2 years, *p* = *0.043*) at the time of operation. There were no statistically significant differences observed in associated anomalies (2.8% vs 3.0%, *p* = *1.000*), intraoperative blood loss (38.0 ml vs. 34.0 ml, *p* = *0.701*), operative time (403 min vs. 414 min, *p* = *0.750*), postoperative complication rate (11.4% vs. 10.9%, *p* = *1.000*), or length of hospital stay (10 days vs. 10 days, *p* = *0.769*) between the two groups. ESSQ surgeons had significantly more years of experience (26 years vs. 16 years, *p* < *0.001*). The mean number of surgeries performed by ESSQ surgeons during the study period was 10.7, while that of surgeries performed by non-ESSQ surgeons was 2.6.

**Table 2:** Demographics and operative outcomes of choledochal cyst patients undergoing surgery performed by qualified and nonqualified surgeons based on the JSES Endoscopic

Factors	ESSQ		Non-ESSQ	*p value*
Patients (n)	36		65	
Conversion to open surgery (n)	0		0	
Male/Female (n/n)	10/26		14/51	*0.480*
Body weight at operation (kg)*	19.3 [9.7–48.5]		12.1 [8.9–18.0]	***0.031***
Age (y)*	5 [1–15]		2 [1–6]	***0.031***
Associated anomalies (n)	1 (2.8%) Malrotation		2 (3.0%) Duodenal atresia with malrotation, Cardiac anomaly	*1.000*
Blood loss (ml)*	41.5 [13.3–78.3]		31.0 [11.5–78.0]	*0.622*
Operative time (min)*	398 [344–447]		414 [336–478]	*0.807*
Postoperative complication (n)	4 (11.1%)		7 (10.8%)	*1.000*
Length of hospital stay (day)*	10 [8–14]		10 [8–13]	*0.647*
Years of experience (y)	26 [20–29]		16 [9–20]	** < *0.001***

^*^Median (range)

Table 3 summarizes the comparison between ESSQ and non-ESSQ groups for lobectomy. One case in the non-ESSQ group was converted to open surgery towing to intraoperative ventilatory failure, this case was excluded from the statistical analysis. No statistically significant differences were observed in body weight of the patients at the time of operation (7.6 kg vs. 7.1 kg, *p* = *0.512*), age of patients (0 years vs. 0 years, *p* = *0.220*), intraoperative blood loss (5.0 ml vs. 5.0 ml, *p* = *0.703*), or operative time (179 min vs. 175 min, *p* = *0.714*) between the two groups. The postoperative complication rate was numerically higher in the ESSQ group compared to the non-ESSQ group (12.9% vs. 2.9%), although this difference did not reach statistical significance (*p* = *0.179*). Moreover, no associated anomalies were observed in lobectomy. ESSQ surgeons had significantly more years of experience (24 years vs. 16 years, *p* < *0.001*). The mean number of surgeries performed by ESSQ surgeons during the study period was 7.5, while that of surgeries performed by non-ESSQ surgeons was 2.1.

**Table 3:** Demographics and operative outcomes of lobectomy patients undergoing surgery performed by qualified and nonqualified surgeons based on the JSES Endoscopic

Factors	ESSQ	Non-ESSQ	*p value*
Patients (n)	31	36	
Conversion to open surgery (n)	0 (0%)	1 (2.8%)	*1.000*
Male/Female (n/n)	15/16	17/18	*0.988*
Body weight at operation (kg)*	7.6 [4.1–13.1]	7.1 [5.8–8.6]	*0.512*
Age (y)*	0 [0–3]	0 [0–0]	*0.220*
Associated anomalies (n)	0	0	*-*
Blood loss (ml)*	5.0 [1.0–40.0]	5.0 [1.0–40.0]	*0.703*
Operative time (min)*	179 [136–260]	175 [144–218]	*0.714*
Postoperative complication (n)	4 (12.9%)	1 (2.9%)	*0.179*
Years of experience (y)	24 [21–28]	16 [6–19]	** < *0.001***

^*^Median (range)

## Discussion

This study analyzed data from a total of 246 pediatric advanced endoscopic surgeries at a single center in Japan. Our findings demonstrated that advanced MIS can be safely performed with good postoperative outcomes by young pediatric surgeons in the learning phase when supervised by an ESSQ-qualified pediatric surgeon. This is comparable to open abdominal or thoracic surgery [6]. In adult surgery, some reports indicate that the ESSQ qualification is associated with shorter operating times and less blood loss, indicating superior perioperative outcomes for ESSQ-qualified surgeons [7–11]. However, other reports indicate no significant differences in postoperative complications, mortality, or hospital stay, suggesting that non-ESSQ-qualified surgeons can also perform operations safely under the supervision of ESSQ-qualified surgeons [12–15]. There are few reports comparing young surgeons with limited experience and skilled surgeons with well-developed experience in pediatric surgery. For esophageal atresia, ESSQ qualification results in shorter operative times but no significant difference in postoperative complications [16]. However, no other studies have examined the feasibility of endoscopic surgery for young pediatric surgeons with such a substantial number of cases.

This study indicated that young pediatric surgeons can safely perform MIS under the supervision of ESSQ-qualified surgeons. However, the complex nature of surgical outcomes is influenced by multiple factors. The shorter operative times observed for BA in the ESSQ group suggests that personal experience in specific types of endoscopic surgery can impact surgical outcomes. However, the nonsignificant differences in postoperative outcomes for either condition suggest that surgical technique alone does not fully explain patient outcomes. Factors such as patient demographics, disease severity, and perioperative management protocols may influence postoperative complications.

The differences in patient characteristics between the ESSQ and non-ESSQ groups observed for CC in this study suggest that ESSQ-qualified surgeons may be allocated more challenging cases. This highlights the need for comprehensive patient selection criteria and standardized procedural protocols to reduce potential confounding variables. The ESSQ-qualified surgeons also decided which patients would be treated by the younger surgeons. Patient selection, alongside surgical technique, seems crucial for young surgeons to perform procedures safely. When an ESSQ-qualified judges that a patient can be safely operated on by a young surgeon, the young surgeon feels confident in performing the procedure ensuring postoperative results comparable to those of an ESSQ-qualified surgeon. Notably, our findings do not guarantee that complicated patients can be safely operated on by surgeons without ESSQ qualification.

Ongoing efforts to standardize training programs and strengthen mentoring will play a pivotal role in ensuring young pediatric surgeons maintain high standards of patient care [17]. Moreover, non-ESSQ-qualified surgeons in our training program have extensive surgical experience, having completed three years of adult surgical training before specializing in pediatric surgery. Even non-ESSQ-qualified surgeons have a certain degree of surgical experience. Pediatric surgery involves deals with many rare diseases, and we have developed disease-specific simulators and actively provide off-the-job training [18, 19]. Non-ESSQ-qualified surgeons at our institution are required to achieve a certain skill level through dry box training before performing endoscopic surgeries.

This study contributes to the ongoing debate regarding the role of young surgeons and the potential impact of an aging surgical workforce. The number of surgeons in Japan is declining, with the number of young surgeons having dropped by half compared to 20 years ago [20]. In this context, the importance of conveying the appeal of surgery, enhancing the clinical education curriculum, and providing early exposure to surgical experience has been advocated [21]. Early exposure to pediatric surgery is often challenging due to the limited number of cases and the specialized skills required compared to adult surgery. While it is essential to provide opportunities for young surgeon, maintaining a balance that ensures patient safety and optimal outcomes.

Children, in particular, deserve the highest standards of care, which inexperienced surgeons alone may not be able to guarantee. Therefore, supervision by experienced surgeons is necessary for procedures to be performed safely and effectively. Our findings suggest that early exposure to pediatric surgery is feasible through careful preoperative planning, supervision by skilled surgeons, and attention to patient safety. Regarding future of pediatric surgery, such exposure will be beneficial for the career development of individuals, ensuring a skilled workforce, promoting skill development, and ultimately improving patient outcomes. Further research, including prospective studies and more comprehensive assessments of surgeons’ skills, will help us better understand the dynamics between experienced and inexperienced surgeons in pediatric surgery.

There may be some limitations to this study. First, as a retrospective study with a limited sample size at a single center, there may be biases in the data and its generalizability to other medical centers may be limited. Second, although this study focused primarily on perioperative outcomes, the time period was limited to 2013–2023. Further investigation is needed to assess the long-term impact on patient outcomes. Third, the study relies on the ESSQ certification system to evaluate the skills of surgeons. Notably, not all skilled endoscopic surgeons are certified, and it does not necessarily follow that noncertified surgeons do not have superior skills. However, at our institution, all skilled endoscopic surgeons are ESSQ qualified.

This study compared pediatric surgeons with and without ESSQ certification. While significant differences were found only in operative time for biliary atresia, no significant differences were observed in other perioperative outcomes for biliary atresia, choledochal cyst, or lobectomy. These results indicate that young, non-ESSQ-certified pediatric surgeons can safely perform minimally invasive surgery under the supervision of experienced ESSQ-certified surgeons.
